# Use of wireless telephones and self-reported health symptoms: a population-based study among Swedish adolescents aged 15–19 years

**DOI:** 10.1186/1476-069X-7-18

**Published:** 2008-05-21

**Authors:** Fredrik Söderqvist, Michael Carlberg, Lennart Hardell

**Affiliations:** 1 Department of Oncology, University Hospital, Institute of Clinical Medicine, Örebro University, SE-701 85 Örebro, Sweden; 2 Department of Oncology, University Hospital, SE-701 85 Örebro, Sweden

## Abstract

**Background:**

Despite the last years of rapid increase in use of wireless phones little data on the use of these devices has been systematically assessed among young persons. The aim of this descriptive cross-sectional study was to assess use of wireless phones and to study such use in relation to explanatory factors and self-reported health symptoms.

**Methods:**

A postal questionnaire comprising 8 pages of 27 questions with 75 items in total was sent to 2000 Swedish adolescents aged 15–19 years and selected from the population registry using a stratified sampling scheme.

**Results:**

The questionnaire was answered by 63.5% of the study subjects. Most participants reported access to a mobile phone (99.6%) and use increased with age; 55.6% of the 15-year-olds and 82.2% of the 19-year-olds were regular users. Girls generally reported more frequent use than boys. Use of wired hands-free equipment 'anytime' was reported by 17.4%. Cordless phones were used by 81.9%, and 67.3% were regular users. Watching TV increased the odds ratio for use of wireless phones, adjusted for age and gender. Some of the most frequently reported health complaints were tiredness, stress, headache, anxiety, concentration difficulties and sleep disturbances. Regular users of wireless phones had health symptoms more often and reported poorer perceived health than less frequent users.

**Conclusion:**

Almost all adolescence in this study used a wireless phone, girls more than boys. The most frequent use was seen among the older adolescents, and those who watched TV extensively. The study further showed that perceived health and certain health symptoms seemed to be related to the use of wireless phones. However, this part of the investigation was explorative and should therefore be interpreted with caution since bias and chance findings due to multiple testing might have influenced the results. Potentially this study will stimulate more sophisticated studies that may also investigate directions of associations and whether, or to what degree, any mediation factors are involved.

## Background

The use of wireless telephones such as mobile and cordless phones (DECT) is increasing rapidly worldwide. The mobile phone penetration rate in many European countries today exceeds 100% (subscribers per hundred inhabitants); in December 2005 Sweden had a penetration rate of 112% [1]. In spite of this development there is little data on the actual use of these devices in the general population. Health concerns related to the use of wireless phones also underline the need recognized by the World Health Organization to document rapidly-changing patterns of wireless communication use [2]. Concerns may pertain either to biological or non-biological effects where the latter is an indirect effect following use of wireless phones rather than a direct effect of emissions.

As for biological effects a recent review on possible effects of radio frequency fields on human health concludes that no such effect has been consistently shown at exposure levels below the limits of the International Committee on Non-Ionising Radiation (ICNIRP). However, the evaluation of long-term exposure remains limited, and in respect of the latter, mobile phone use by children should receive special attention as children and teenagers of today will experience a much higher cumulative exposure than previous generations [3]. Another concern is whether children are more sensitive to the exposure than adults. There are theoretical reasons to be concerned [4] but insufficient scientific grounds to generally condemn mobile phone use by children [5].

When we began our study in 2005 only one systematic study had been published with the aim of surveying ownership and use of wireless phones among young persons [6]. Since then several studies have been published [7-12], and four of these measured some sort of health aspects in relation to use [8-10,12]. According to some of the findings intensive mobile phone use may be part of the same health-related lifestyle as health compromising behaviors and was more common among those with less privileged social background. In one study intensive use also seemed to be gender-specific as it formed a risk for girls' perceived health mostly explained through deteriorated sleeping habits [9]. In another study it was reported that mobile phone use after lights out was associated with increased risk of self-reported tiredness after one year of follow up [12].

In addition to the few existing reports on adolescents several studies have investigated associations between mobile phone use and self-reported health symptoms among adults.

In epidemiological studies headache has been one of the most frequently reported symptoms [13-15], but it should also be mentioned that more recently conducted provocation studies have failed to demonstrate a convincing causal link [16-19]. On the other hand one could also argue that a provocation study does not account for possible long-term effects, only acute effects.

We report here the results of wireless telephone use among Swedish adolescents. This is a continuation of a previous study, which included 7–14-year-olds; a descriptive cross-sectional study that used the same methodology [11]. More specifically the aims of the study reported in this paper were (1) to assess use of wireless phones and to determine any age or gender differences in such use, (2) to examine factors related to regular use of such phones and (3) to explore the association between use of wireless phones and self-reported health symptoms. Because of limitations in the study design, the ambition in respect of the third aim was first and foremost to generate a hypothesis. We were interested in whether frequent use of wireless phones was in any way related to certain more frequently reported health symptoms or to perceptions of health, as described e.g. by Punamäki et al [9].

## Methods

### Collection of data

The local ethics committee approved the study methods. We used a stratified sampling scheme to recruit study subjects so that for each age group in the 15–19 years range, 200 boys and 200 girls were randomly selected from the Swedish population registry. In total, 2000 individuals were selected for the study. The population registry, which contains information on current municipal residency, was used to link each subject's living area code to a so-called homogeneity region, classified by Statistics Sweden. This officially lists six different regions (H1–H6) all categorized by population density and the number of inhabitants in the vicinity of the main city in that municipality [20], for more details, see Söderqvist et al [11]. To collect our data we used a specially designed questionnaire that was sent, along with a letter of information, to each subject's guardian for subjects in the age group 15–17 years and directly to subjects aged 18–19 years. All questionnaires (n = 2000) were mailed to the study subjects during October 2005 to be returned by July 2006 (n = 1269). Supplementary questions were sent by mail to improve the quality of data when necessary. Subjects who had not returned their questionnaires after two reminders were regarded as non-responders.

The questionnaire comprised 8 pages of 27 questions with 75 items in total. The respondents were asked to answer the questions either by ticking the appropriate alternative, or if none of these matched to write the answer in free text, e.g. average use of mobile phone in minutes per day. The first 9 questions concerned background data such as age, sex, age of guardian, income of household etc. Then followed questions concerning the use of various wireless devices such as mobile phones, DECT, and wireless Internet connections at home or in school, wireless earphones and other wireless music equipment. Three different types of mobile phones were assessed: the digital 3G- (third generation mobile phone), GSM-phones (global system for mobile communication) and the analogue NMT-phones (Nordic Mobile Telephone).

Questions were asked about TV-watching, sleep habits and physical activity. The questions addressed twenty different types of physical activities as well as the number of hours spent per week in three categories: 1–7, 8–14 or > 14 hours. An open question was also included in case the respondent's activity was not among those listed. The information from these questions was then used to analyse factors that could explain regular use of mobile phones and DECT, two outcome variables by which the distribution of the study base was classified. Regular mobile phone use was defined as talk ≥ 2 min/day and regular DECT use as talk ≥ 5 min/day. Finally, the respondents were asked to fill out a list of health symptoms. They were asked if they had experienced each symptom, and if so, how often they suffered from it: 'never', 'seldom', 'every week' or 'every day'. Occurrence of these symptoms was therefore based on subjective evaluation by each individual – whether they had had the symptom and if so how frequently – and not on medical records. The questionnaire ended with a question concerning the respondents' perception of health during the last two months. The alternatives to choose from were 'very good', 'good' 'quite good', 'poor' or 'very poor'.

### Statistical methods

Frequency tables were produced for all variables. Questions relating to the aim of the study were chosen for further analysis to determine any age or gender differences in mobile phone or DECT use and whether there was any statistically significant association between use of wireless phones and reported health symptoms or perceived health. Questions concerning differences between groups in use of wireless devices were first examined by χ^2 ^tests. We then used unconditional logistic regression analysis for further calculation of odds ratios (OR) and 95% confidence intervals (CI) for factors that could explain regular mobile phone and DECT use. We adjusted for age and sex since these variables were significantly associated with regular mobile phone and DECT use according to the χ^2 ^test. Dependent variables for this analysis were regular mobile phone use/no regular use and regular DECT use/no regular use. Independent variables were explanatory factors such as H-regions, the existence of siblings, overweight condition and obesity, time spent watching TV, time spent playing computer games and amount of physical activity. Use of DECT was also included as an independent variable to predict use of mobile phone and vice versa (see Table 2).

**Table 2:** Odds ratios (OR) and 95% confidence intervals (CI) for factors that could explain regular use of mobile phone and DECT.

	Adolescents who reported regular mobile phone use*			Adolescents who reported Regular DECT use**		
	Exposed/Unexposed	OR	95% CI	Exposed/Unexposed	OR	95% CI

Household income						
Average	368/191	1.0	-	304/154	1.0	-
< Average	106/33	1.4	0.9 – 2.2	81/31	1.3	0.8 – 2.0
> Average	312/139	1.2	0.9 – 1.6	250/126	1.0	0.8 – 1.4
H-regions						
H1	149/56	1.0	-	125/44	1.0	-
H2	122/46	1.0	0.6 – 1.6	105/36	1.0	0.6 – 1.7
H3	330/160	0.8	0.5 – 1.1	268/130	0.7	0.5 – 1.1
H4	149/88	0.6	0.4 – 0.95	128/77	0.6	0.4 – 0.9
H5	60/20	1.1	0.6 – 2.1	42/28	0.5	0.3 – 0.97
H6	56/19	1.1	0.6 – 2.0	27/22	0.5	0.2 – 0.9
Siblings						
No	41/11	1.0	-	28/11	1.0	-
Yes	825/378	0.6	0.3 – 1.1	667/326	0.8	0.4 – 1.7
Overweight						
No	721/314	1.0	-	565/266	1.0	-
Yes	114/57	0.9	0.6 – 1.2	103/56	0.9	0.6 – 1.4
Obesity						
No	808/361	1.0	-	640/312	1.0	-
Yes	27/10	1.0	0.5 – 2.2	28/10	1.2	0.6 – 2.6
Time spent watching TV						
< 30 min per day	87/54	1.0	-	71/41	1.0	-
≥ 30 – 60 min per day	228/103	1.5	0.99 – 2.3	174/96	1.2	0.7 – 2.0
> 60 – 180 min per day	390/185	1.4	0.97 – 2.1	337/144	1.6	1.02 – 2.5
> 180 min per day	148/38	2.4	1.4 – 4.0	105/52	1.2	0.7 – 2.0
Time spent playing computer games						
Never	404/130	1.0	-	325/94	1.0	-
< 30 min per day	177/89	0.8	0.5 – 1.1	143/78	0.8	0.5 – 1.1
≥ 30 – 60 min per day	105/65	0.7	0.5 – 1.1	76/67	0.6	0.4 – 0.95
> 60 – 180 min per day	113/66	0.8	0.5 – 1.3	88/64	0.8	0.5 – 1.3
> 180 min per day	65/37	0.8	0.5 – 1.4	60/33	1.2	0.7 – 2.0
Physical activity						
1–7 hours per week	440/220	1.0	-	348/183	1.0	-
8–14 hours per week	164/70	1.3	0.9 – 1.8	138/60	1.5	1.02 – 2.1
> 14 hours per week	33/10	1.4	0.7 – 3.0	23/12	1.0	0.4 – 2.0
Use of DECT						
Never	137/70	1.0	-			
< 5 min per day	166/163	0.6	0.4 – 0.9			
≥ 5 – 15 min per day	244/90	1.5	1.04 – 2.3			
> 15 – 30 min per day	170/40	2.4	1.5 – 3.7			
> 30 min per day	130/17	4.1	2.3 – 7.4			
Mobile phone access						
< 2 min per day				147/163	1.0	-
≥ 2 min – 5 min per day				159/75	2.3	1.6 – 3.4
> 5 min – 15 min per day				217/55	3.8	2.6 – 5.6
> 15 min – 30 min per day				89/17	5.0	2.8 – 9.0
> 30 min – 60 min per day				46/12	3.3	1.7 – 6.6
> 60 min per day				33/7	3.9	1.6 – 9.2

* = Defined as talking ≥ 2 min per day – related to those who claim to have mobile phone access.

** = Defined as talking ≥ 5 min per day – related to those who claim to have DECT access.

Unconditional logistic regression analysis adjusted for age and gender was used. Numbers of 'exposed' (regular use) and 'unexposed' (no regular use) in the different categories are shown.

Overweight condition and obesity were defined according to age and gender, as suggested by Cole et al., assuming BMI over 25 as overweight and over 30 as obesity in adults [21]. Physical activity was classified into three groups according to number of hours per week. Adjustments were made for age as a continuous variable and for income by using three categories with the average income group as reference (OR = 1.0). Family income was defined as suggested by The Ratio Institute, a trade research institute [22]. Below average was defined as < 200,000 SEK per year, average family income as 200,000–450,000 SEK per year and above average family income as > 450,000 SEK per year.

To estimate associations between regular use of wireless phones, health symptoms and perceived health ordinal logistic regression adjusted for age and sex was used. The ordinal health symptoms variables (with the alternatives 'never', 'seldom', 'every week', 'every day') and perceived health ('very good', 'good' 'quite good', 'poor', 'very poor') were dependent variables in the analyses. Unconditional logistic regression adjusted for age and sex was used to calculate OR and 95% CI for associations between regular use of wireless phones and perceived insufficient sleep since the latter was included as a separate question and not included in the symptoms list. No correction for multiple endpoints was made for either of the analyses. Subjects reporting no use or no regular use of mobile phone and DECT were regarded as unexposed (Tables 3, 4, 5). For all statistical analyses, Stata 8.2 was used (Stata/SE 8.2 for Windows; StataCorp, College Station TX).

**Table 3:** Odds ratios (OR) and 95% confidence intervals (CI) for self-reported health symptoms (no symptom, seldom, every week, every day) and use of mobile phone.

	**Total mobile phone use**		**≥ 2 min – 15 min per day**		**> 15 min per day**	
	OR	CI	OR	CI	OR	CI

1. Allergic symptoms	1.3	0.96–1.8	1.2	0.9–1.7	1.6	1.1–2.4
2. Asthmatic symptoms	1.8	1.1–3.0	1.8	1.03–3.0	2.0	1.1–3.6
3. Other breathing difficulties	1.1	0.7–1.9	1.1	0.6–1.8	1.4	0.8–2.4
4. Chest pain	0.9	0.6–1.4	0.8	0.5–1.3	1.1	0.7–1.9
5. Palpitation	1.3	0.8–2.1	1.2	0.7–2.0	1.5	0.8–2.6
6. Hay fewer	1.4	0.9–2.0	1.3	0.9–2.0	1.6	1.01–2.5
7. Eczema	1.3	0.9–1.9	1.2	0.8–1.9	1.3	0.8–2.1
8. Dizziness	1.4	0.96–2.0	1.3	0.9–1.9	1.6	1.1–2.5
9. Headache	1.5	1.1–2.0	1.5	1.1–2.0	1.6	1.2–2.3
10. Anxiety	1.2	0.9–1.6	1.2	0.9–1.6	1.3	0.9–1.9
11. Concentration difficulties	1.4	1.1–1.9	1.4	1.02–1.8	1.6	1.1–2.3
12. Depressed mood	1.0	0.7–1.3	1.0	0.7–1.3	1.1	0.8–1.6
13. Sleep Disturbances	1.1	0.8–1.4	1.0	0.8–1.4	1.2	0.9–1.7
14. Stress	1.3	0.98–1.7	1.2	0.9–1.6	1.6	1.1–2.2
15. Tiredness	1.3	0.98–1.7	1.2	0.9–1.6	1.5	1.04–2.0
16. Cold sweat	1.2	0.8–1.8	1.1	0.7–1.6	1.5	0.9–2.4
17. Skin rash	1.4	0.9–2.1	1.3	0.9–2.0	1.5	0.95–2.5
18. Tingling/burning sensation of the skin	1.1	0.7–1.7	1.0	0.7–1.6	1.3	0.8–2.2
19. Eye irritation	1.0	0.7–1.4	0.9	0.7–1.3	1.2	0.8–1.8
20. Tinnitus	0.9	0.7–1.3	0.8	0.6–1.2	1.3	0.8–1.9
21. Body pain	1.1	0.8–1.5	1.1	0.8–1.5	1.2	0.8–1.8
22. Pricking sensation in the mouth	1.7	0.7–4.1	1.4	0.6–3.6	2.4	0.9–6.4
23. Often catch infections	1.1	0.7–1.6	1.1	0.7–1.6	1.1	0.7–1.8

Ordinal logistic regression analysis adjusted for age and gender was used.

**Table 4:** Odds ratios (OR) and 95% confidence intervals (CI) for self-reported health symptoms (no symptom, seldom, every week, every day) and use of DECT.

	**Total DECT use**		**≥ 5 min – 15 min per day**		**> 15 min per day**	
	OR	CI	OR	CI	OR	CI

1. Allergic symptoms	1.4	0.98–1.9	1.3	0.9–1.9	1.4	0.97–2.0
2. Asthmatic symptoms	1.9	1.1–3.3	2.2	1.3–3.8	1.7	0.9–3.0
3. Other breathing difficulties	1.2	0.7–2.0	1.0	0.6–1.8	1.5	0.9–2.5
4. Chest pain	1.0	0.7–1.6	0.9	0.6–1.5	1.1	0.7–1.8
5. Palpitation	1.4	0.8–2.3	1.5	0.9–2.7	1.2	0.7–2.2
6. Hay fewer	1.5	1.01–2.2	1.5	0.9–2.2	1.5	1.002–2.4
7. Eczema	1.3	0.9–2.0	1.2	0.8–1.9	1.4	0.9–2.2
8. Dizziness	1.4	0.99–2.1	1.1	0.7–1.6	1.8	1.2–2.8
9. Headache	1.5	1.2–2.1	1.2	0.9–1.7	2.0	1.5–2.8
10. Anxiety	1.2	0.9–1.6	1.1	0.8–1.6	1.3	0.96–1.9
11. Concentration difficulties	1.4	1.03–1.9	1.2	0.9–1.6	1.6	1.2–2.2
12. Depressed mood	1.1	0.8–1.4	1.0	0.7–1.4	1.2	0.8–1.6
13. Sleep Disturbances	1.1	0.8–1.5	1.0	0.7–1.4	1.2	0.9–1.7
14. Stress	1.4	1.03–1.8	1.1	0.8–1.5	1.7	1.3–2.4
15. Tiredness	1.3	1.01–1.8	1.3	0.97–1.8	1.4	0.99–1.9
16. Cold sweat	1.2	0.8–1.8	1.1	0.7–1.7	1.3	0.8–2.0
17. Skin rash	1.4	0.9–2.1	1.5	0.9–2.3	1.3	0.8–2.1
18. Tingling/burning sensation of the skin	1.2	0.8–1.9	1.1	0.7–1.8	1.3	0.8–2.1
19. Eye irritation	1.1	0.8–1.5	1.0	0.7–1.5	1.2	0.8–1.8
20. Tinnitus	1.0	0.7–1.4	0.8	0.5–1.2	1.2	0.8–1.7
21. Body pain	1.2	0.9–1.6	1.0	0.7–1.4	1.4	0.997–2.0
22. Pricking sensation in the mouth	2.0	0.8–5.0	1.9	0.7–5.0	2.2	0.8–5.7
23. Often catch infections	1.1	0.7–1.7	1.1	0.7–1.7	1.2	0.7–1.9

Ordinal logistic regression analysis adjusted for age and gender was used.

**Table 5:** Odds ratios (OR) and 95% confidence intervals (CI) for self-reported perceived health (very good, good, fair, poor, very poor) and use of mobile phone or DECT. Ordinal logistic regression analysis adjusted for age and gender was used.

	**Total**		**≥ 2 min – 15 min per day (mobile phone)/≥ 5 min – 15 min per day (DECT)**		**> 15 min – 30 min per day**		**> 30 min per day**	
	OR	CI	OR	CI	OR	CI	OR	CI

Mobile phone								
Perceived health	1.3	1.01–1.7	1.2	0.9–1.6	1.7	1.1–2.6	1.8	1.2–2.7
DECT								
Perceived health	1.3	1.002–1.8	1.2	0.9–1.7	1.3	0.9–1.8	1.7	1.1–2.5
Mobile phone*								
Perceived health	1.1	0.9–1.5	1.1	0.8–1.4	1.4	0.9–2.1	1.5	0.97–2.3
DECT*								
Perceived health	1.1	0.8–1.5	1.1	0.8–1.5	1.1	0.8–1.6	1.4	0.9–2.1

* = Adjusted for insufficient sleep and tiredness.

## Results

The participation rate was 63.5% (n = 1269) of which 52.2% were girls. No trend of differences in response rate was found with regard to population density or gender. The percentage of missing data among the participants was highest for questions on background characteristics: 3.4% for weight, 3.7% for age of father and 3.7% for household income. Missing data on questions related to use of wireless telephones did not exceed one percent.

**Figure 1:**
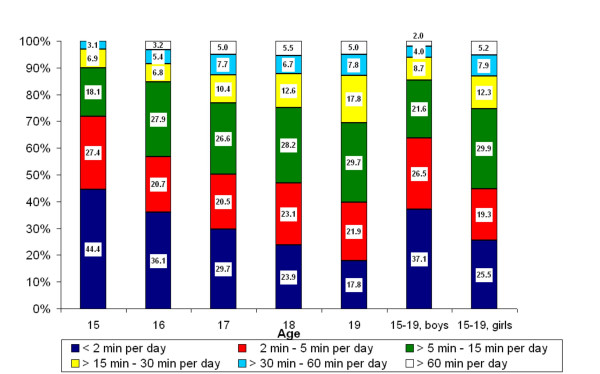
Percentage distributions of average mobile phone use according to age and gender.

Overall, 99.6% of the respondents (99.8% girls, 99.3% boys) reported that they had access to a mobile phone; 81.9% used a digital GSM-phone, 16.2% a 3G-phone and 0.7% the analogue NMT-phone. Figure 1 displays the reported average mobile phone use in minutes (min) per day, by age and gender. The data clearly show that use increased with age. For example, when regular mobile phone use (≥ 2 min/day) was analysed, the percentage of such users among the 15-year-olds was 55.6% while among the 19-year-olds it was 82.2%.

Use of wired hands-free equipment was reported by 17.4% of those claiming mobile phone access; 0.5% used it always, 3.2% often, 13.5% less frequently and 0.2% did not specify usage. Frequency of hands-free use increased with age, from 14.8% among the 15-year-olds to 24.1% among the 19-year-olds. Wireless hands-free equipment use was reported by 3.9%. Sending and receiving SMS and MMS was common among respondents with mobile phone access; 62.3% reported sending ≥ 1 or more times a day and there was no significant gender difference.

**Figure 2:**
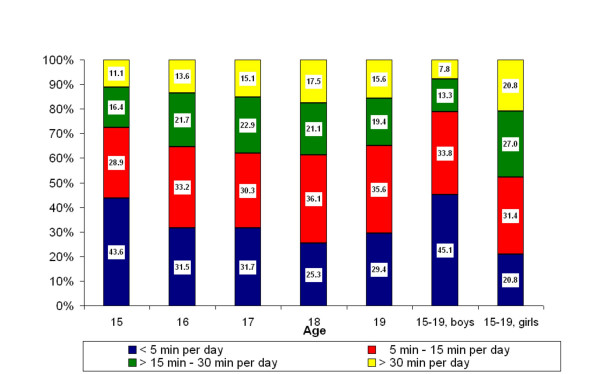
Percentage distributions of average cordless phone use according to age and gender.

Overall, 94.6% of the respondents reported having a landline phone at home; 12.5% had only a phone with wire, 37.6% only a cordless phone and 44.3% had both. In Figure 2, DECT use in the different age groups is shown; comparison with Figure 1 shows that DECT users used the phone for more minutes per day than mobile phone users. However, the trend of increasing use with age was not as evident for DECT as for mobile phones. Of the respondents 81.9% reported use of DECT and 67.3% were regular users.

Use of wireless music equipment was reported by 4.7% of the respondents, wireless earphones for music listening by 3.6% and walkie-talkie by 3.9%.

The data in Table 1, Figure 1 and Figure 2 show that gender as well as age was associated with more frequent use of wireless phones. Girls reported more regular mobile phone use than boys, 74.5% versus 62.9%. The corresponding results for regular use of DECT were 79.2% versus 54.9%. A gender difference was also seen for reported use of wired hands-free equipment (20.7% girls, 13.8% boys), and for sending and receiving SMS ≥ 1 per day (73.8% by girls, 49.9% by boys) (data not in table).

**Table 1:** Factors that could explain regular mobile phone use and regular DECT use among 15–19 years old subjects in Sweden.

	Adolescents who reported regular mobile phone use*			Adolescents who re-ported regular DECT use**		
	Total in category	%	N	Total in category	%	N

Age (in years)						
15	259	55.6	(144)	225	56.4	(127)
16	280	63.9	(179)	235	68.5	(161)
17	259	70.3	(182)	218	68.3	(149)
18	238	76.1	(181)	194	74.7	(145)
19	219	82.2	(180)	160	70.6	(113)
p, χ^2^-test		<0.001			0.001	
Sex						
Female	659	74.5	(491)	529	79.2	(419)
Male	596	62.9	(375)	503	54.9	(276)
p, χ^2^-test		< 0.001			< 0.001	

* = Defined as talking ≥ 2 min per day – related to those who claim to have mobile phone access.

** = Defined as talking ≥ 5 min per day – related to those who claim to have DECT access.

A statistically significant association was found between population density region and regular use of DECT. As the Table 2 shows, living in sparsely populated areas such as regions H4–H6 yielded OR = 0.6 or less and 95% CI that did not encompass unity. Time spent watching TV gave increased OR for regular use of both mobile phone and DECT. A regular mobile phone user was more likely than a non-regular user to be a regular DECT user, and vice versa.

The most frequently reported health complaints were tiredness, stress, headache, anxiety, concentration difficulties and sleep disturbances. Overall, girls reported higher scores than boys on all self-reported health symptoms. The self-reported health symptoms were analysed in relation to mobile phone use and the results are shown in Table 3. Regular use of mobile phone (total use) yielded significantly increased OR for asthmatic symptoms (OR = 1.8, 95% CI = 1.1–3.0), headache (OR = 1.5, 95% CI = 1.1–2.0) and concentration difficulties (OR = 1.4, 95% CI = 1.1–1.9). Dividing regular mobile phone use into two groups, ≥ 2 – 15 min and > 15 min per day, increased the OR further. The same was seen for most symptoms. Borderline significant associations were found for allergic symptoms, dizziness, stress and tiredness in total. Adjusting for use of hands-free equipment (n = 200) did not change the results. The corresponding results for use of DECT are shown in Table 4. As for mobile phones regular use of DECT (total use) gave a significantly increased OR for asthmatic symptoms (OR = 1.9, 95% CI = 1.1–3.3) and headache (OR = 1.5, 95% CI = 1.2–2.1). Borderline significant associations were found for allergic symptoms, hay fever, dizziness, concentration difficulties, stress and tiredness in the total. When the reported health symptoms were analysed in relation to mobile phone or DECT use, no statistically significant gender differences were seen (data not in table). Besides the 23 health symptoms we also analyzed insufficient sleep as a separate question. Regular use of mobile phones gave OR = 1.9, 95% CI = 1.3–2.6 and regular use of DECT OR = 1.9, 95% CI = 1.4–2.7. For both phone types ORs increased with use in min per day with some gender differences.

Concerning perception of health during the last two months, 32.7% perceived their health as 'very good', 37.3% as 'good', 23.3% as 'fair', 5.3% as 'poor' and 1.4% as 'very poor'. There were statistically significant gender differences in perceived health: girls reported poorer health than boys (P < 0.001). For example, 42.0% of the boys reported 'very good' health compared with 24.1% of the girls, and 3.9% of the boys reported bad or very bad health compared with 9.3% of the girls. Perceived health was also analysed in relation to use of wireless phones; the results are summarized in Table 5. No significantly increased ORs were found for regular use of mobile phones and DECT in a further analysis adjusting for insufficient sleep and tiredness although ORs increased with minutes of use per day. We also performed gender-specific analyses of perceived health as presented in Table 5, and the results among regular users did not differ significantly between girls and boys (data not shown).

## Discussion

This study showed that use of mobile phones and DECT increased with age so that almost all adolescents used wireless phones. Comparing the use of the two phone types by age in Figure 1 and Figure 2 showed that DECT accounted for the most frequent use while the increase in use by age was more evident for mobile phones. Gender differences were seen; girls used mobile phones and DECT significantly more than boy as also reported in three Finnish studies [8-10]. In our study the largest difference was seen for use of DECT with twice as many girls than boys that reported regular use. Use of a hands free device was generally low but has not been assessed in other studies.

Time spent watching TV increased the OR for wireless phone use, especially mobile phones as also reported in our previous study [11] and in a German study [6]. This can perhaps be explained by a combination of age and lifestyle factors. For example, older adolescents are probably more likely to be able to afford use of a mobile phone frequently and to stay up late to watch TV. It is also in this group of users one would expect the mobile phone to be used more as an immediate communication tool for social networking and to deepen friendships, compared with younger groups. Decreased OR for use of DECT was found in sparsely populated areas, which indicates that this technology is more widely adopted in more populated areas, e.g. with people living in apartments. No such trend was found for use of mobile phones. Unfortunately no information was assessed on frequency of use of a wired phone so we cannot exclude that phone use in general is less popular in the sparsely populated areas. Household income, overweight condition, obesity, spent time playing computer games and amounts of physical activity were not significantly related to use of mobile phones or DECT, after adjustment for age and gender.

To study health complaints in relation to use of wireless phones 23 self-reported symptoms were assessed and certain health symptoms were reported more frequently among regular mobile phone users. Significantly increased ORs for total mobile phone use (regular use compared with no use or no regular use of mobile phone and DECT) were found for asthmatic symptoms, concentration difficulties and headache. For most symptoms ORs increased further in the group of individuals with regular mobile phone use of more than 15 minutes per day. Similar results were found for regular use of DECT, but use of these phone types was significantly intercorrelated (p < 0.001). If these results were an effect of exposure they could either have been the direct effect of emissions or an indirect effect following the use of a wireless phone, such as for example tiredness or deteriorated sleeping habits. The latter has recently been reported in two studies [9,12], one in which it was shown that mobile phone use after lights out was related to increased levels of tiredness [12]. High levels of wireless phone use might increase the likelihood that the wireless phone is also used after lights out, which leads to tiredness and concentration difficulties during the day. This in turn might lead to e.g. increased frequency of headaches. In one analysis of wireless phone use and headache we adjusted for insufficient sleep, which did not change the results. Unfortunately no information on wireless phone use after lights out was obtained so this matter cannot be adequately solved within this investigation.

Gender differences were seen both with regard to reported health complaints and perceived health: girls suffered more frequently from health symptoms and reported poorer perceived health than boys. One likely explanation for these differences is that women might have different and broader preferences than men when making general ratings of health, such as psychological factors and health symptoms [23]. However, since the girls in this study also reported more frequent use of wireless phones than boys we performed gender-specific analyses for health symptoms as presented in Table 3 and 4, which did not yield higher ORs for girls than for boys (data not shown). Thus, while being a girl was in itself a "risk factor" for health problems and poor perceived health, using a mobile phone or a DECT did not increase the risk more for girls than for boys.

The literature on the use of wireless phones and the associations with health symptoms and perceived health in adolescence is sparse. Two studies familiar to us are those reported by Koivusilta et al. [8] and Punamäki et al. [9]. The latter is only partly comparable with our investigation because of differences in how health was assessed. In our study we looked at self-reported symptoms and perceived health separately in relation to use of wireless phones, whereas Punamäki et al. [9] used a combined variable for health. With regard to use of mobile phones in relation to health the Finnish studies gave similar results: frequent mobile phone use was associated with poor perceived health, although in one of the studies mediated through deterioration of sleeping habits and increased waking-time tiredness [9]. Adjustments for insufficient sleep and tiredness had only a small effect in our study. Other similar differences were also seen with regard to gender as in our study.

Recently conducted provocation studies have failed to demonstrate a convincing causal link between any reported symptoms and exposure to mobile phone like signals [16-19]. Evidence of a causal relationship thus seems unlikely for acute effects, which is the main focus of provocation studies. Epidemiological studies, however, may also include possible long-term effects not detectable in provocation studies. Longitudinal cohort studies are of course preferable to cross-sectional designs, but also expensive and time-consuming. One also has to consider the delicate issue of assessing exposure over time as the technology changes.

Our study had several limitations; its cross-sectional design was one. Another was the assessment of the use of wireless phones, which was not validated by e.g. billing records. The main reason why we chose not to undertake such validation was that pay-as-you-call use of mobile phones is quite frequent in the studied age group and is not recorded. Another problem with using billing records is that incoming calls are not recorded. Regarding use of DECT, the problem is obviously how to discriminate between such use and use of a wired phone. One way to validate estimates could be to use specially software-modified phones handed out to a sample of the respondents, but an even better method might be to use a personal dosimeter, preferably one that detects a wide band of exposure frequencies.

The relatively low response rate (63.5%) could also have biased the results. We did compare early responders with those sent at least one reminder and found no statistically significant difference (p = 0.53) in average use of mobile phones. Nor did we find such a difference when we compared the percentage of regular users of mobile phones (p = 0.14) or DECT (p = 0.79). Self-reporting of health symptoms may be another limitation. It would have been desirable to obtain medical records to verify certain complaints like for instance concentration difficulties and stress whereas other symptoms are more obvious e.g. asthma, hay fewer and eczema.

As for the significant associations found for wireless phone use and health symptoms we have reasons to suspect that these may not have been an effect of exposure or random error, but instead influenced by systematic error, since for both use of mobile phone and DECT there were only a few ORs below 1. That could have been due to confounding e.g. by a socio-economic factor or perhaps bias due to previous opinions among the respondents as a result of the way the questionnaire was designed. There is some information that indicates more frequent use of mobile phone in non-nuclear families and among adolescents whose fathers have low education or socioeconomic status [8]. We adjusted for household income used as a proxy for SES, which did not change the results nor did adjustment for watching TV (data not shown). Further adjustment for use of hands-free equipment was also made for statistically significant associations such as headache, but the results did not change as reported in a study from Singapore [13]. In that study the prevalence of headache was reduced by 20% among those who use hands-free equipment.

Finally we should point out the explorative nature of the results relating to the third aim of this investigation with use of multiple testing for the 23 health symptoms in relation to wireless phone use. By doing so we increased the probability of chance findings – assuming independence between occurrences of these symptoms. Clearly another study design should be used to investigate the directions of associations, if any. We should also add that no information about traditional health-compromising behaviors was assessed, which might be of importance since in one study smoking and alcohol drinking were reported to correlate with intensive mobile phone use [10].

## Conclusion

In conclusion this study showed that almost all adolescence used a wireless phone, girls more than boys. The most frequent use was seen among the older adolescents, and those who watched TV extensively. The reported use of hands-free equipment was low. The study further showed that perceived health and certain health symptoms seemed to be related to the use of wireless phones. However, this part of the investigation was explorative and should therefore be interpreted with caution since bias and chance findings due to multiple testing might have influenced the results. Potentially this study will stimulate more sophisticated studies that may also investigate directions of associations and whether, or to what degree, any mediation factors are involved.

## List of abbreviations

CI: Confidence Interval; DECT: Digitally enhanced cordless phone; GSM: Global system for mobile communication; H: Homogeneity regions; ICNIRP: International Commission on Non-Ionising Radiation Protection; NMT: Nordic Mobile Telephone System; n: Number; OR: Odds ratio; SMS: Short text message; 3G: Third generation mobile phones

## Competing interests

The authors declare that they have no competing interests.

## Authors' contributions

FS was the principal investigator responsible for design, conduct, analysis, interpretation of data and writing the manuscript.

MC participated as statistician and in the compilation and interpretation of the data for this publication

LH made contributions to conception and design and also to analysis and drafting the manuscript.
